# Spironolactone Ameliorates Senescence and Calcification by Modulating Autophagy in Rat Tendon-Derived Stem Cells via the NF-*κ*B/MAPK Pathway

**DOI:** 10.1155/2021/5519587

**Published:** 2021-06-30

**Authors:** Kai Xu, Changjian Lin, Diana Ma, Mengyao Chen, Xing Zhou, Yuzhe He, Safwat Adel Abdo Moqbel, Chiyuan Ma, Lidong Wu

**Affiliations:** ^1^Department of Orthopedic Surgery, The Second Affiliated Hospital, Zhejiang University School of Medicine, Hangzhou City, Zhejiang Province, China; ^2^Orthopedics Research Institute of Zhejiang University, Hangzhou City, Zhejiang Province, China; ^3^Key Laboratory of Motor System Disease Research and Precision Therapy of Zhejiang Province, Hangzhou City, Zhejiang Province, China; ^4^Department of Medical Oncology, The 2nd Affiliated Hospital, Zhejiang University School of Medicine, Hangzhou, China

## Abstract

Tendinopathy is a disabling musculoskeletal disease, the pathological process of which is tightly associated with inflammation. Spironolactone (SP) has been widely used as a diuretic in clinical practice. Recently, SP has shown anti-inflammatory features in several diseases. Tendon-derived stem cells (TDSCs), a subset cell type from tendon tissue possessing clonogenic capacity, play a vital role in the pathological process of tendinopathy. In the present study, the protective effect of SP on TDSCs was demonstrated under simulated tendinopathy conditions both *in vitro* and *in vivo*. SP contributed to the maintenance of TDSC-specific genes or proteins, while suppressing the interleukin- (IL-) 1*β*-induced overexpression of inflammation-mediated factors. Additionally, IL-1*β*-induced cellular senescence in TDSCs was inhibited, while autophagy was enhanced, as determined via *β*-galactosidase activity, western blot (WB), and quantitative real-time polymerase chain reaction analysis. With the aid of several emerging bioinformatics tools, the mitogen-activated protein kinase (MAPK) pathway likely participated in the effect of SP, which was further validated through WB analysis and the use of MAPK agonist. Immunofluorescence analysis and an NF-*κ*B agonist were used to confirm the inhibitory effect of SP on IL-1*β*-induced activation of the NF-*κ*B pathway. X-ray, immunofluorescence, immunohistochemistry, hematoxylin and eosin staining, histological grades, and Masson staining showed that SP ameliorated tendinopathy in an Achilles tenotomy (AT) rat model *in vivo*. This work elucidates the protective role of SP on the pathological process of tendinopathy both *in vitro* and *in vivo*, indicating a potential therapeutic strategy for tendinopathy treatment.

## 1. Introduction

Tendinopathy is a chronic, degenerative condition characterized by a failed healing response within the tendon tissue [1], which accounts for a significant portion of visits to sports medicine doctors. Despite its socioeconomic and health burden, the underlying mechanisms of tendinopathy remain elusive. Recent studies indicate that inflammation is involved in tendon homeostasis, as well as the resolution of tendon damage [2, 3]. A number of investigators have demonstrated that proinflammatory mediators such as interleukin 1*β* (IL-1*β*) initiate fibrosis [4], extracellular matrix degradation, and apoptosis [5], eventually leading to tendinopathy. Hence, we hypothesized that modulation of inflammation might be a potential therapeutic target for tendinopathy. Heterotopic ossification (HO) is another hallmark of tendinopathy [6, 7]. It has been proposed that HO plays a significant role in the pathological progression of tendinopathy [8]. Nonetheless, neither the underlying mechanism nor effective treatments for calcium deposition are fully understood [9].

Tendon-derived stem cells (TDSCs) are isolated subsets of cell populations from tendon tissues and possess clonogenic capacity. With a high proliferation rate and multipotency, functional TDSCs may play a dominant role in tendon maintenance and healing. An increasing number of studies have focused on TDSCs rather than tenocytes to investigate tendinopathy. Recently, researchers reported that inhibition of cellular inflammation in TDSCs contributes to the amelioration of tendinopathy [10, 11]. For example, aspirin, a cyclooxygenase (COX) inhibitor, inhibits tendinopathy and decreases rerupture risk of injured tendons by suppressing inflammation [12]. Based on the evidence above, repressing TDSC inflammation might be a potential therapeutic strategy for treating tendinopathy.

SP, which pharmacologically binds cytoplasmic mineralocorticoid receptors and functions as an aldosterone antagonist, has been widely used in the treatment of ascites, nephrotic syndrome, and congestive heart failure [13]. Recently, extensive evidence has linked SP to its anti-inflammatory effects; for example, Mortensen et al. have reported that SP reduces vascular inflammation in patients with renal transplants [14], while Zhang et al. have demonstrated that SP is capable of preventing peritoneal fibrosis and inflammation in patients undergoing peritoneal dialysis [15]. However, it is currently unknown whether SP intervention would significantly inhibit TDSC inflammation in the tendinopathic process. The protective effect of SP on tendinopathy has not yet been reported.

In the present study, a series of experiments were designed to investigate the positive effect of SP on tendinopathy both *in vitro* and *in vivo*. Several emerging bioinformatics tools were used to predict the underlying mechanism involved in SP function, which was further validated by substantial experiments.

## 2. Materials and Methods

### 2.1. Cell Culture

This study was approved by the Institutional Animal Care and Use Committee of Zhejiang University (Hangzhou, China). Achilles tendons were obtained from 3-week-old Sprague Dawley rats (Zhejiang Academy of Medical Sciences, Hangzhou, China) and cut into 1 mm^3^ particles. Tendons were then incubated with 3 mg/mL type I collagenase on a horizontal shaker at 37°C for 3 h to isolate tenocytes. Single-cell tendon-derived cells were cultured in 96-well plates for 7 d, and colonies were collected as passage 0 (P0) and passaged three times prior to use in all experiments. Dulbecco's modified Eagle medium (DMEM) supplemented with 10% feral bovine serum (FBS), 100 units/mL penicillin, and 100 *μ*g/mL streptomycin was used to expand single-cell colonies. Cells were cultured at 37°C with 5% CO_2_.

### 2.2. Reagents

Spironolactone was purchased from Sigma, USA (CAS50-01-7). DMEM, FBS, streptomycin, penicillin, and 0.25% pancreatic enzyme were all obtained from Gibco, NY, USA. Recombinant rat IL-1*β* was purchased from R&D Systems (Abingdon, UK), and collagenase II, dimethyl sulfoxide, and bovine serum albumin (BSA) were obtained from Sigma-Aldrich (St. Louis, MO, USA). The bicinchoninic acid assay kit and radioimmunoprecipitation assay (RIPA) buffer were purchased from Beyotime Institute of Biotechnology (Shanghai, China). Asiatic acid and betulinic acid were purchased from Selleck Chemicals.

### 2.3. Identification of Trilineage Differentiation Potential

Cells were incubated in specific differentiation media. For osteogenesis, cells were incubated in osteogenic induction medium (Cyagen Biosciences) for 14 d, and Alizarin Red staining was used to confirm the differentiation to osteoblasts. For adipogenesis, cells were incubated in adipogenic induction and maintenance medium (Cyagen Biosciences) for 14 d, and Oil Red O staining was used to confirm the differentiation to adipocytes. For chondrogenesis, cells were incubated in pellet culture with chondrogenic induction medium (Cyagen Biosciences) for 21 d, and Safranin O staining was used to confirm the chondrocyte differentiation.

### 2.4. Flow Cytometry

Cells were incubated with fluorescent primary antibody on ice in phosphate-buffered saline (PBS) for 60 min, washed three times, and detected using flow cytometry. The negative control contained no fluorescent antibodies. The following fluorescent primary antibodies were used: fluorescein isothiocyanate (FITC) anti-rat CD29, FITC anti-rat CD44, phycoerythrin (PE) anti-rat CD45, and PE anti-rat CD90 (Bioleague).

### 2.5. Cell Viability Analysis

To analyze the cytotoxicity of SP on TDSCs, a Cell Counting Kit-8 (CCK-8) assay was conducted according to the manufacturer's instructions. Cells (5 × 10^3^) were seeded into 96-well plates and treated with different concentrations of SP (0, 0.1, 1, 10, 50, and 100 *μ*M) for 48 h. Cells were incubated with 10 *μ*L CCK-8 reagent per well for 3 h, and then, the absorbance was read at a wavelength of 450 nm with a microplate spectrophotometer.

### 2.6. Immunofluorescence

TDSCs cultured on 24-well plates were pretreated with SP (0, 0, and 10 *μ*M) for 1 h and then incubated with IL-1*β* (10 ng/mL) for 30 min. After fixation with methanol for 30 min, the cells were permeabilized with PBS containing 0.5% *v*/*v* Triton X-100 for 15 min and blocked with 5% BSA for 1 h. The cells were incubated with primary antibody against p65 at 4°C overnight, followed by incubation with FITC-conjugated secondary antibodies for 1 h. Cell nuclei were stained with 4′,6-diamidino-2-phenylindole (DAPI) for 5 min, and then, cells were analyzed using a Leica fluorescence microscope.

Tendon sections were incubated with the primary antibody against RUNX family transcription factor 2 at 4°C overnight. The sections were then incubated with a FITC-conjugated secondary antibody. Nuclei were stained with DAPI, according to the manufacturer's instructions. The results were visualized by fluorescence microscopy.

### 2.7. Quantitative Real-Time Polymerase Chain Reaction (qRT-PCR)

Total RNA was extracted using the TRIzol® Plus RNA Purification Kit (Invitrogen, Carlsbad, CA, USA). The concentrations of RNA were detected and adjusted using nucleic acid detector before reverse transcription with PrimeScript™ RT Master Mix (Takara). cDNA samples were replicated with SYBR® Premix Ex Taq™ II (Takara) with an Applied Biosystems StepOnePlus™. The expression of scleraxin (*Scx*), mohawk homeobox (*Mkx*), tenomodulin (*Tnmd*), inducible nitric oxide synthase (*iNOS*), cyclooxygenase 2 (*COX2*), matrix metalloproteinase 9 (*MMP9*), *MMP13*, autophagy related 5 (*ATG5*), *ATG7*, and Beclin-1 was detected. Glyceraldehyde 3-phosphate dehydrogenase (*GAPDH*) was used as an endogenous control. The primers used are listed in Table 1. All of the above experiments were performed in triplicate according to the manufacturer's instructions using three independent samples. The data were calculated using the 2(−*ΔΔ*CT) method.

### 2.8. *β*-Galactosidase Activity Assay

The *β*-galactosidase activity assay (Beyotime Biotechnology, Shanghai, China) was performed to measure cellular senescence according to the manufacturer's instructions. Cells were cultured in *β*-galactosidase staining buffer for 24 h and visualized under a microscope.

### 2.9. Reactive Oxygen Species (ROS) Detection

TDSCs cultured on 24-well plates were pretreated with SP (0, 0, and 10 *μ*M) for 1 h and then incubated with IL-1*β* (10 ng/mL) for 24 h. A ROS assay kit (Beyotime) was used to detect intracellular ROS in TDSCs, according to the manufacturer's instructions. The fluorescence intensity of the intracellular ROS was visualized using fluorescence microscopy and quantitated using ImageJ.

### 2.10. Western Blot (WB) Analysis

After treatment, TDSCs were washed three times with PBS and lysed with RIPA buffer for 60 min. Then, the samples were separated via 10% or 15% sodium dodecyl sulfate polyacrylamide electrophoresis and transferred onto nitrocellulose membranes. The membranes were blocked with 5% BSA for 1 h and cut into sections based on different protein molecular weights. The membranes were incubated with primary antibodies at 4°C overnight. Then, the membranes were incubated with secondary antibodies for 1 h and luminescence was determined using the Pierce™ ECL western blotting substrate. The relative amount of proteins was analyzed using Quantity One software (Bio-Rad) and normalized to GAPDH. All assays were performed in triplicate.

### 2.11. Animal Model

Eighteen male Sprague Dawley rats (200-250 g; 6 weeks old) were randomly divided into three groups (six rats per group): negative control (NC), Achilles tenotomy (AT), and AT+SP. Rats in the NC group underwent sham surgery, and the other rats underwent Achilles tenotomy. One week after Achilles tenotomy or sham surgery, the AT+SP group was injected with 0.1 mL of 10 ng/mL SP once a week in the region surrounding the Achilles tendon, and the AT group was injected with 0.1 mL vehicle. Nine weeks after Achilles tenotomy or sham surgery, the rats were sacrificed for X-ray and histological analyses.

### 2.12. Histological Analysis

The lower limb samples were cut into 5 *μ*m sections deparaffinized with xylene and subsequently rehydrated using a graded ethanol series. These sections were stained with hematoxylin and eosin (HE) and Masson staining, according to the manufacturer's instructions. Histological scores were calculated from HE staining results [16].

### 2.13. Immunohistochemistry Analysis

Immunohistochemical staining was used to assess tendinopathy in the sagittal sections of the limb from each group. Limb sections were prepared as described in the histological analysis and then subjected to antibodies against MMP9 (Abcam, ab76003).

### 2.14. X-Ray

An X-ray machine was used to evaluate the calcifications of the Achilles tendon. Lateral X-ray images of the legs of the rats were generated at 60 kV with a radiation intensity of 500 mA (200 Ma).

### 2.15. Statistical Analysis

All data are presented as the mean ± standard deviation (SD). One-way analysis of variance with a subsequent post hoc Tukey's test was used for multiple comparisons. Statistical significance was set at *p* < 0.05.

## 3. Results

### 3.1. Isolation and Characterization of TDSCs

**Figure 1:**
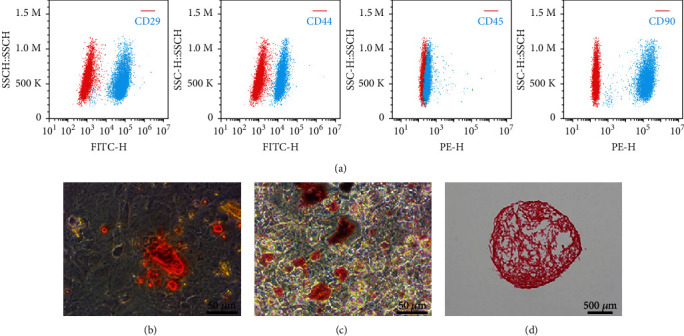
Identification of TDSCs. (a) Representative flow cytometric images of TDSCs stained with CD29, CD44, CD45, and CD90 (red: control; blue: fluorescent antibody). Trilineage differentiation of TDSCs: (b) osteogenesis (Alizarin Red); (c) adipogenesis (Oil Red O); (d) chondrogenesis (Safranin O).

To ascertain the clonogenicity of the isolated cells, a cell surface marker analysis was performed. Flow cytometry results showed that these cells were positive for the stem/precursor cell markers CD29, CD44, and CD90, but not for the leukocyte marker CD45 [17, 18] (Figure 1(a)). The multidifferentiation potential of the putative TDSCs toward chondrogenesis, adipogenesis, and osteogenesis was then identified. Alizarin Red staining of 2-week-old osteogenic cultures showed calcium deposits in the cell culture (Figure 1(b)). Oil Red O staining of 2-week-old adipogenic cultures showed accumulation of lipids within the cells (Figure 1(c)). Safranin O staining of 3-week-old chondrogenic cultures determined the chondrolineage differentiation of TDSCs (Figure 1(d)). SP had no significant effect on osteogenic, adipogenic, and chondrogenic differentiation of TDSCs (Supplementary Figure 1).

### 3.2. SP Contributed to the Maintenance of Phenotype in TDSCs

**Figure 2:**
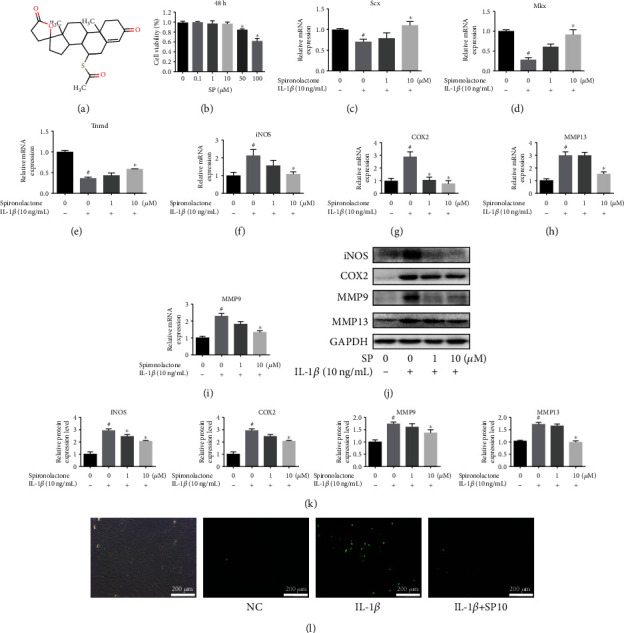
SP attenuated IL-1*β*-induced inflammation in TDSCs. (a) Molecular structural formula of SP. (b) CCK-8 analysis of treated TDSCs. (c–f) qRT-PCR was performed to analyze the relative mRNA expression of iNOS, COX2, MMP13, and MMP9. (g) Results of western blot analysis and (h–k) quantitative analysis of corresponding proteins: iNOS, COX2, MMP9, and MMP13. (l) Representative immunofluorescence images of ROS in treated TDSCs (green: ROS antibody). ^#^*p* < 0.05 versus the control group. ^∗^*p* < 0.05 versus the model group.

The molecular structure of SP is shown in Figure 2(a). To further assess the cytotoxicity of SP on TDSCs, the CCK-8 assay was performed in a dose-dependent manner (0.1, 1, 10, 50, and 100 *μ*M). As shown in Figure 2(b), SP has no obvious cytotoxicity in TDSCs until the concentration reaches 50 and 100 *μ*M. Concentrations of 1 and 10 *μ*M were chosen as suitable SP concentrations for subsequent experiments, and 10 ng/mL IL-1*β* was chosen to induce the *in vitro* tendinopathic model [12, 19]. Rat TDSCs express marker genes, such as *Scx*, *Tnmd*, and *Mkx* [17, 20, 21]. qRT-PCR was performed to measure the expression of *Scx*, *Mkx*, and *Tnmd* in IL-1*β*-induced TDSCs and demonstrated that 10 ng/mL IL-1*β* significantly decreased the relative mRNA expression of these marker genes, while SP at an appropriate concentration (10 *μ*M) reversed this effect (Figures 2(c)–2(e)).

### 3.3. IL-1*β*-Induced Inflammation in TDSCs Was Attenuated by SP

To further investigate the effect of SP on IL-1*β*-induced TDSC inflammation, WB, qRT-PCR, and immunofluorescence were performed. qRT-PCR (Figures 2(f)–2(i)) and WB (Figures 2(j) and 2(k)) analysis revealed that SP decreased the expression of universally acknowledged inflammation-related mediators including *iNOS*, *COX2*, *MMP13*, and *MMP9* at the mRNA and protein levels. ROS in treated TDSCs were detected using an immunofluorescence assay, which demonstrated that SP inhibited the IL-1*β*-induced increase in ROS in TDSCs (Figure 2(l)). In conclusion, SP attenuated IL-1*β*-induced inflammation and intracellular ROS levels in TDSCs.

### 3.4. SP Alleviated IL-1*β*-Induced Senescence in TDSCs

**Figure 3:**
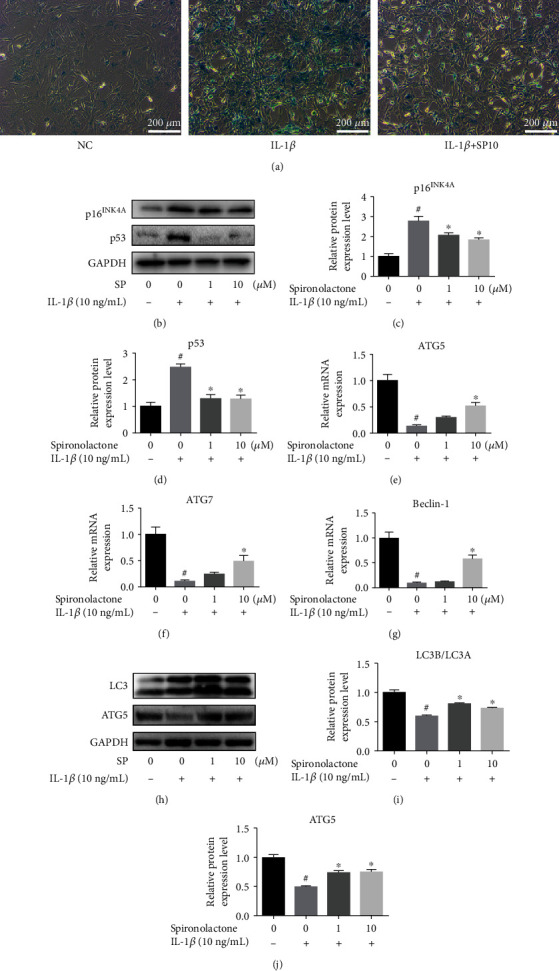
SP ameliorated IL-1*β*-induced senescence while enhanced autophagy in TDSCs. (a) *β*-Galactosidase activity assay was performed in TDSCs treated with or without IL-1*β*/SP10. (b–d) Western blot was performed to analyze the relative protein expression of p16 and p53 in treated TDSCs. (e–g) TDSCs cultured on 6-well plates were pretreated with SP (0, 1, and 10 *μ*M) for 1 h and then incubated with IL-1*β* (10 ng/mL) for 24 h. qRT-PCR analysis of the mRNA expression levels of ATG5, ATG7, and Beclin-1 in treated TDSCs. (h–j) The relative protein expression of LC3 and ATG5 was detected by western blot. ^#^*p* < 0.05 versus the control group. ^∗^*p* < 0.05 versus the model group.

TDSCs cultured on 6-well plates were pretreated with SP (0, 0, and 10 *μ*M) for 1 h and then incubated with IL-1*β* (10 ng/mL) for 24 h. After treatment, a *β*-galactosidase activity assay was conducted (Figure 3(a)). IL-1*β* stimulation activated cellular senescence in TDSCs, while SP reversed this effect. The effect of SP on senescence in TDSCs was further confirmed at the protein level. p16(Ink4a) is involved in cell cycle regulation. Currently, p16(Ink4a) is considered a tumor suppressor protein and appears to be one of the principal factors in senescence [16]. p53 is another crucial protein in senescence, and its activation modulates cellular senescence and organismal aging [22]. WB analysis revealed that the expression of the prosenescence proteins p16^INK4a^ and p53 decreased after SP treatment (Figures 3(b)–3(d)).

### 3.5. SP Enhanced Autophagy in TDSCs

Some studies have shown that autophagy has a protective effect, promoting the survival of tenocytes [23]. In this study, the effect of SP on autophagy of tenocytes was evaluated. qRT-PCR analysis of autophagy biomarkers *ATG5*, *ATG7*, and *Beclin-1* is shown in Figures 3(e)–3(g). IL-1*β* stimulation resulted in the occurrence of autophagic flux, a decrease in the protein expression of *ATG5*, *ATG7*, and *Beclin-1*, and an increase in the protein expression of light chin 3A (LC3A), while intervention with SP reduced the inhibition. Additionally, the same conclusion was obtained through WB analysis of LC3B/LC3A and ATG5 at the protein level (Figures 3(h)–3(j)).

### 3.6. The Mitogen-Activated Protein Kinase (MAPK) Pathway Was Involved in the Effect of SP on TDSCs

**Figure 4:**
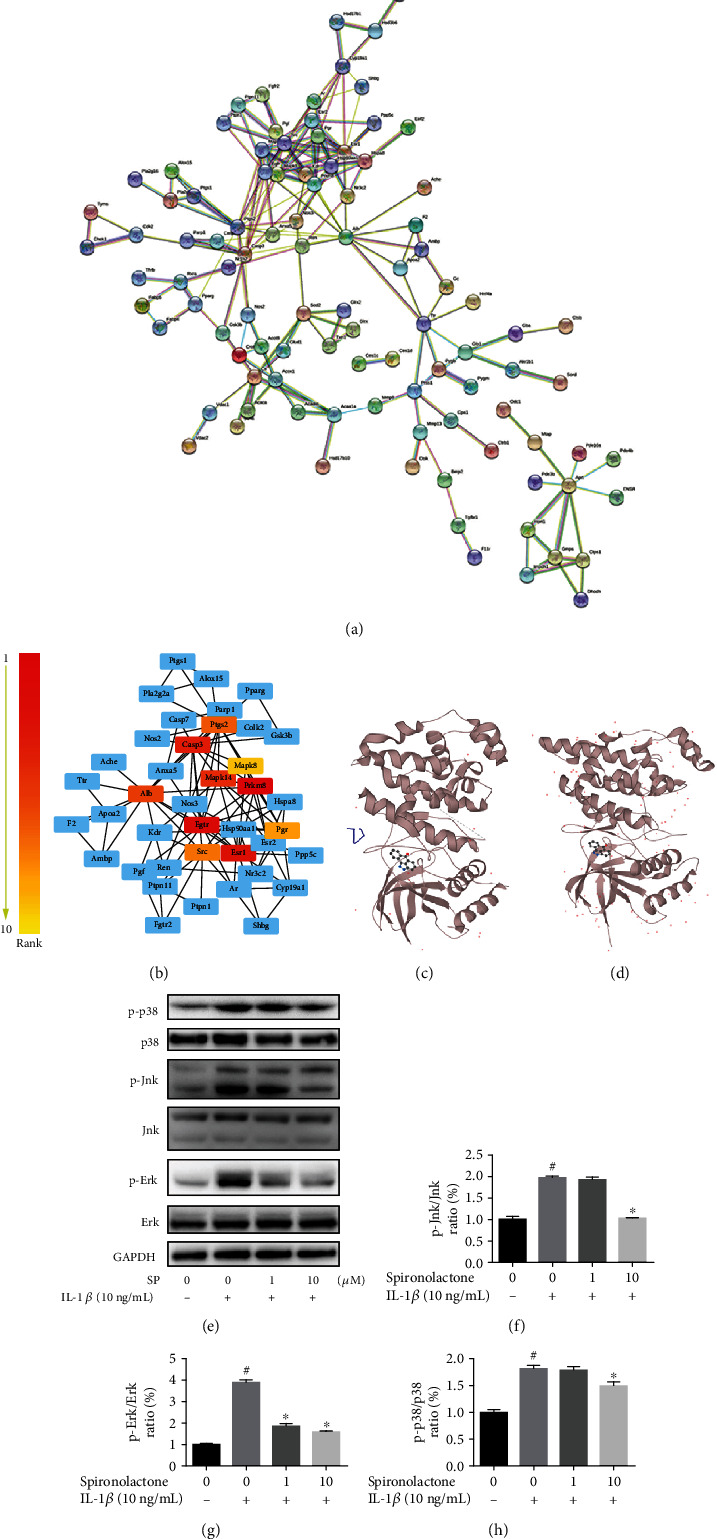
Significant impact of SP on IL-1*β*-induced MAPK activation in TDSCs. TDSCs cultured on 6-well plates were pretreated with SP (0, 1, and 10 *μ*M) for 1 h and then incubated with IL-1*β* (10 ng/mL) for 30 minutes. After treatment, a series of western blot analysis were performed to detect the activation of the MAPK pathway. (a) PharmMapper was used to obtain the potential targets of SP. Subsequently, the protein-protein association network of potential SP targets was established via STRING and shown in (a). (b) The hub genes from the network were predicted and calculated by Cytoscape. Top 10 hub genes were marked as shown in the figure. We used the database UniProt to draw the predictive 3D binding diagrams of (c) SP and MAPK8 and (d) SP and MAPK10. (e) WB analysis of MAPK pathway-related proteins: p-p38, p38, p-Jnk, Jnk, p-Erk, Erk, and internal reference GAPDH. The ratio of phosphorylated proteins/proteins: (f) p-Jnk/Jnk, (g) p-Erk/Erk, and (h) p-p38/p38. ^#^*p* < 0.05 versus the control group. ^∗^*p* < 0.05 versus the model group.

To elucidate the downstream mechanism of SP function, potential SP targets were first sought using the web tool PharmMapper [24–26]. The Search Tool for the Retrieval of Interacting Genes/Proteins (STRING) database was used to establish protein-protein association networks from the potential SP targets obtained from PharmMapper [27]. An integrated model constructed using Cytoscape [28] is shown in Figure 4(a). Hub genes have been calculated in Cytoscape using the cytoHubba plug-in, and their ranks are shown in Figure 4(b). MAPK8, MAPK14, and other MAPK-related proteins were identified in the top 10 hub genes. The predictive three-dimensional binding models of SP and MAPK8 (Figure 4(c)) and SP and MAPK10 (Figure 4(d)) have been drawn using UniProt [29]. These bioinformatics predictions suggested that SP exerted its function through the MAPK pathway. To confirm this speculation, WB analysis was conducted. The ratio of p-c-Jun N-terminal kinase (Jnk)/Jnk, p-extracellular signal-regulated kinase (Erk)/Erk, and p-p38/p38 increased after treatment with SP (Figures 4(e)–4(h)). Thus, IL-1*β* activated the MAPK pathway, while SP inhibited this activation, as expected. In summary, this work demonstrated that SP functions in IL-1*β*-induced TDSCs by suppressing MAPK pathway activation.

### 3.7. Effect of SP on IL-1*β*-Induced NF-*κ*B Activation in TDSCs

**Figure 5:**
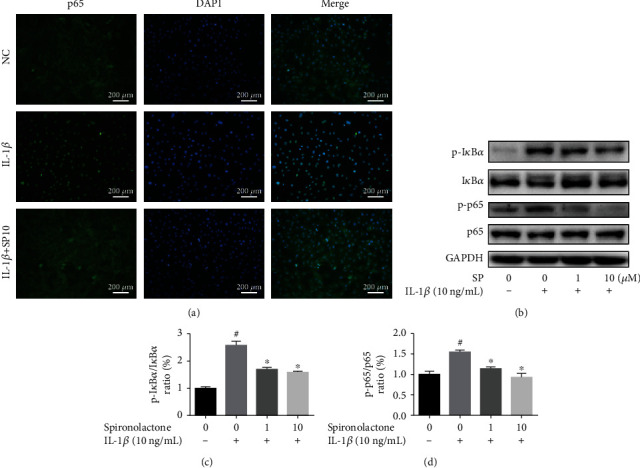
SP influenced the NF-*κ*B pathway in TDSCs. (a) Representative images of p65 nuclear translocation through immunofluorescence analysis. Blue: DAPI; green: p65. (b) TDSCs cultured on 6-well plates were pretreated with SP (0, 1, and 10 *μ*M) for 1 h and then incubated with IL-1*β* (10 ng/mL) for 30 minutes. Western blot analysis of NF-*κ*B pathway-related proteins: p-I*κ*B*α*, I*κ*B*α*, p-p65, and p65. The ratio of relative protein expression of (c) p-I*κ*B*α*/I*κ*B*α* and (d) p-p65/p65. ^#^*p* < 0.05 versus the control group. ^∗^*p* < 0.05 versus the model group.

Representative images of immunofluorescence staining showed that SP treatment blocked IL-1*β*-induced p65 translocation (Figure 5(a)). WB analysis was conducted to measure the relative protein expression of biomarkers in the NF-*κ*B pathway: p-I*κ*B*α*, I*κ*B*α*, p-p65, and p65 (Figure 5(b)). The ratio of p-I*κ*B*α*/I*κ*B*α* and p-p65/p65 decreased after treatment with SP (Figures 5(c) and 5(d)). These findings revealed that SP suppressed IL-1*β*-induced activation of NF-*κ*B in TDSCs.

### 3.8. Activation of the MAPK/NF-*κ*B Pathway Reversed the Effects of SP on TDSCs

**Figure 6:**
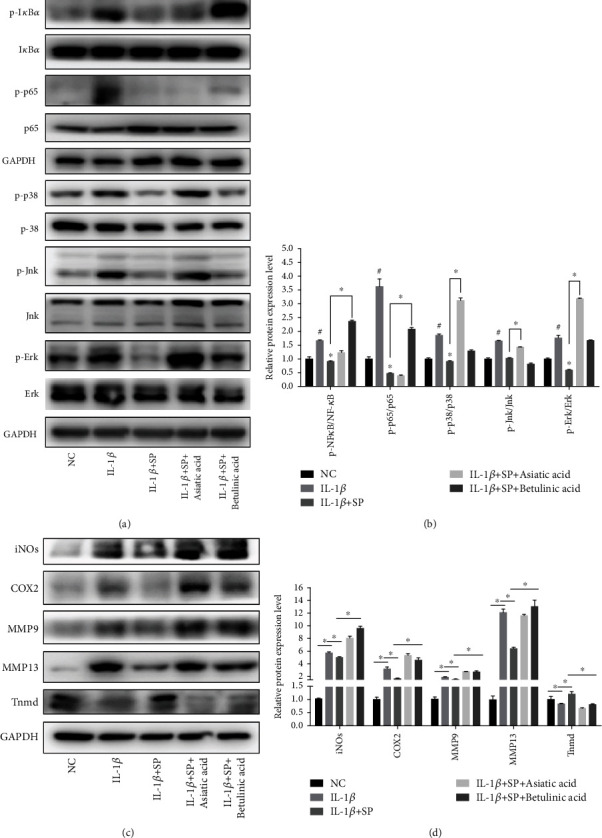
Activation of the MAPK/NF-*κ*B pathway reverse the effects of SP on TDSCs. (a) Western blot analysis of MAPK/NF-*κ*B-related proteins. (b) The ratio of relative protein expression of p-I*κ*B*α*/I*κ*B*α*, p-p65/p65, p-Jnk/Jnk, p-Erk/Erk, and p-p38/p38. (c, d) Western blot analysis of iNOS, COX2, MMP9, MMP13, Tnmd. Asiatic acid: the agonist of the MAPK pathway. Betulinic acid: the agonist of the NF-*κ*B pathway. ^#^*p* < 0.05 versus the control group. ^∗^*p* < 0.05 versus the model group.

To further investigate the role of the MAPK/NF-*κ*B pathway in the effects of SP on TDSCs, the agonist of p38 MAPK, asiatic acid [30], and the agonist of NF-*κ*B, betulinic acid, were used *in vitro* [31]. First, it was confirmed that asiatic acid reactivated the MAPK pathway, while betulinic acid reactivated the NF-*κ*B pathway in SP- and IL-1*β*-treated TDSCs (Figures 6(a) and 6(b)). After treatment with asiatic acid or betulinic acid, WB analysis showed that the relative protein expression of iNOS, COX2, MMP9, and MMP3 increased, while the relative protein expression of Tnmd decreased compared to that of the IL-1*β*+SP group (Figures 6(c) and 6(d)). These results indicated that reactivation of the MAPK/NF-*κ*B pathway reversed the protective effect of SP in an *in vitro* tendinopathic model.

### 3.9. SP Inhibited Calcification and Inflammation In Vivo

**Figure 7:**
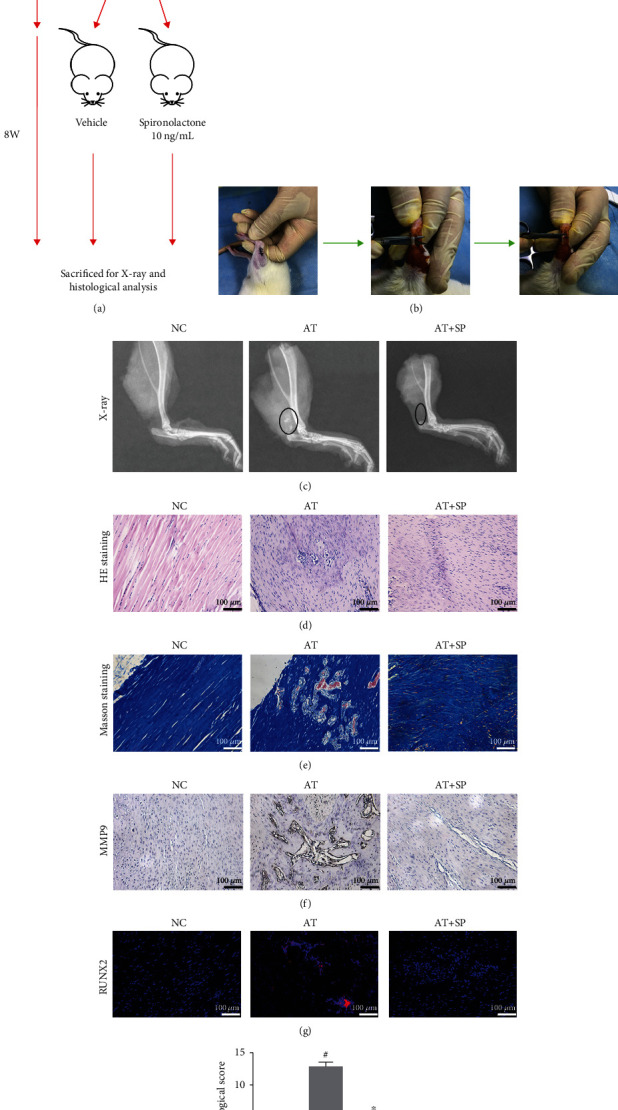
SP inhibits calcification and inflammation in vivo. (a) The flowchart for animal procedures. (b) The diagram of Achilles tenotomy surgery. (c) X-ray was performed to detect the heterotopic ossification. The area within the black circle indicated heterotopic ossification. (d) HE staining of the Achilles tendon in three groups. Bar = 100 *μ*m. (e) Masson staining of the Achilles tendon in three groups. Bar = 100 *μ*m. (f) Representative images of immunohistochemistry using MMP9 antibody. Bar = 100 *μ*m. (g) Representative images of immunofluorescence using RUNX2 antibody. (h) Histological score from HE staining. Blue: DAPI; red: RUNX2. Bar = 100 *μ*m.

To determine the protective role of SP in vivo, an Achilles tenotomy rat model was used to mimic the *in vitro* tendinopathy model [6, 7, 32]. The flowchart for the animal procedures is shown in Figure 7(a). A diagram of Achilles tenotomy is shown in Figure 7(b). X-ray was performed to detect HO in the Achilles tendon area (Figure 7(c)) and demonstrated that the heterotopic bone in the AT group was much larger than that in the NC or AT+SP groups. In the histological analysis, both HE (Figure 7(d)) and Masson staining (Figure 7(e)) showed that SP reversed the disordered arrangement of fibroblasts and collagen fibers in the *in vivo* tendinopathy model. The immunohistochemistry results revealed that SP inhibited inflammation (Figure 7(f)), whereas immunofluorescence images showed that SP ameliorated calcification (Figure 7(g)). Histological scores were calculated from the results of HE staining (Figure 7(h)) [16]. Based on these findings, the protective role of SP was confirmed in an *in vivo* tendinopathy model.

## 4. Discussion

Tendinopathy is a disabling musculoskeletal disorder resulting from an imbalance between self-repairing and chronic disruption, manifesting inflammation, HO, and cellular senescence [2, 3, 7, 8]. Currently, the treatments for tendinopathy are divided into surgical and nonsurgical treatments [33]. Since most nonsurgical treatments for tendinopathy lack sufficient evidence to be recommended for clinical use [34], new nonsurgical approaches are required. In the present study, SP was identified as a potential nonsurgical treatment for tendinopathy.

Traditional studies have used tenocytes as a strategy to investigate tendinopathy [35, 36]. Recent studies have proposed the emerging role of TDSCs in the pathogenesis and pathological progression of tendinopathy [18, 37]. Similar to other stem cells, TDSCs possess self-renewal and multipotent differentiation capacities, indicating their role in tissue repair and regeneration [17]. In this study, Alizarin Red staining, Oil Red O staining, and Safranin O staining were used to validate the trilineage differential capacities of isolated cells. Cell surface markers were detected by flow cytometry confirming that the isolated cells were TDSCs; thus, these cells were used in subsequent experiments.

Chronic inflammation contributes to the initiation and progression of tendinopathy [3, 38–40]. Recent studies have reported the anti-inflammatory effect of SP in multiple diseases [4, 14, 15], and this work demonstrated a similar function in tendinopathy. The IL-1*β*-induced *in vitro* tendinopathic model has been widely recognized in various studies [41]. In the present study, SP had no adverse effect on TDSCs at an appropriate concentration; on the contrary, it contributed to the maintenance of the Scx, Tnmd, and Mkx TDSC phenotype. Furthermore, SP ameliorated IL-1*β*-induced inflammation in TDSCs by inhibiting the expression of iNOS, COX2, MMP9, and MMP13, as well as by diminishing ROS. Taken together, it was demonstrated that SP sustained its anti-inflammatory effect on tendinopathic TDSCs, which further prevented the loss of the TDSC phenotype.

Aging tissue-specific stem cells manifest deficiency in both number and repair capacity, driving the reduced regenerative potential of tissues and contributing to the progress of tendinopathy [42, 43]. Since aging is a potential detrimental factor for tendon homeostasis, the effect of senescence on tendinopathy in TDSCs was investigated [44, 45]. Our previous study reported that senescence in TDSCs is a characteristic of HO and chronic inflammation [6, 7]. Similar to previous studies, more senescent TDSCs were detected under tendinopathic conditions. Additionally, TDSCs in tendinopathy manifest increased expression of senescence-related proteins, p16^INK4a^ and p53 [46]. While senescence appeared in the tendinopathy process, these results showed that SP obtained a fairly good antisenescence function on TDSCs *in vitro* IL-1*β*-induced tendinopathy model.

Recent findings have identified the relevance of autophagy and senescence in multiple diseases [47–49]. Moreover, the autophagy process may be artificially divided into three phases: autophagosome induction and nucleation, autophagosome elongation, and autophagosome maturation and degradation [23]. Hence, autophagy in SP-treated TDSCs warrants further research. In the present study, it was demonstrated that SP intervention might reactivate autophagy in IL-1*β*-treated TDSCs. Collectively, these findings indicated that SP attenuated senescence in TDSCs by modulating autophagy. However, further studies are needed to confirm the precise effect of SP on autophagic flux.

To further illustrate the downstream mechanism of the effect of SP on TDSCs, several emerging bioinformatics tools were used: PharmMapper, STRING, Cytoscape, and UniProt [24–29]. The predictive results indicated that the MAPK pathway was likely involved in the realization of SP function. The MAPK signaling pathway, first elucidated in 1994, regulates cell functions, including proliferation, differentiation, and apoptosis [50]. In view of the close relevance of the MAPK and NF-*κ*B pathways [51, 52] and previous studies on the NF-*κ*B pathway in tendinopathy [53, 54], the effect of SP on the NF-*κ*B pathway in TDSCs was further investigated. Both the MAPK and NF-*κ*B pathways were silenced after SP treatment. Moreover, agonists of the MAPK and NF-*κ*B pathways rescued the effect of SP on IL-1*β*-induced TDSCs, suggesting that SP exerted its function on TDSCs via both the MAPK and NF-*κ*B pathways.

An Achilles tenotomy model was used to examine the potential protective effect of SP on tendinopathy *in vivo* [6, 7]. Histological analysis and *in vivo* results showed that SP ameliorated inflammation and HO and reversed disordered arrangement of fibroblasts and collagen fibers. HO, which refers to the abnormal formation of mature bone within extraskeletal soft tissues, was another pathological feature of tendinopathy [6, 7, 55]. These results identified the positive role of SP in alleviating tendinopathy in an *in vivo* tendinopathic model.

The present study demonstrated the protective role of SP in tendinopathy both *in vitro* and *in vivo*. The effect of SP on tendinopathy was comprehensively investigated by modulating several vital phenotypes, including inhibition of inflammation, calcification, and senescence, while enhancing autophagy. Meanwhile, bioinformatics techniques combined with laboratory experiments provided a classical and practical example to use emerging technical tools. Another highlight of this work is that since SP has long been safe and widely used in the clinic, it seems more plausible to realize the application of SP as a tendinopathy treatment. However, the specific application of SP in patients with tendinopathy requires further research and clinical trials.

## 5. Conclusions

This study showed that SP ameliorated senescence and calcification by modulating autophagy in rat TDSCs via the NF-*κ*B/MAPK pathway, indicating it may be a potential therapeutic strategy for tendinopathy treatment.

## Figures and Tables

**Table 1:** Primer sequences used in this study.

Gene	Forward	Reverse
Scx	AACACGGCCTTCACTGCGCTG	CAGTAGCACGTTGCCCAGGTG
Mkx	TTTACAAGCACCGTGACAACCC	ACAGTGTTCTTCAGCCGTCGTC
Tnmd	TGGGGGAGCAAACACTTCTG	TCTTCTTCTCGCCATTGCTGT
iNOS	CCTACGAGGCGAAGAAGGACAG	CAGTTTGAGAGAGGAGGCTCCG
COX2	GAGAGATGTATCCTCCCACAGTCA	GACCAGGCACCAGACCAAAG
MMP13	GCAAACCCTGCGTATTTCCAT	GATAACCATCCGAGCGACCTTT
MMP9	GCAAACCCTGCGTATTTCCAT	GATAACCATCCGAGCGACCTTT
ATG5	ATTCCAACGTGCTTTACTCTCTATC	AAACCAAATCTCACTAACATCTTCT
ATG7	GTGTACGATCCCTGTAACCTAACCC	CGAAAGCAGAGAACTTCAACAGACT
Beclin-1	CGTGGAGAAAGGCAAGATTGAAGA	GTGAGGACACCCAAGCAAGACC
GAPDH	GAAGGTCGGTGTGAACGGATTTG	CATGTAGACCATGTAGTTGAGGTCA

## Data Availability

The datasets generated for this study are available upon request to the corresponding author.

## Supplementary Materials

Supplementary Materials Supplementary Figure: SP has no significant effect on TDSC trilineage differentiation. SP with the concentration of 10 *μ*M/mL. Safranin O staining and Oil Red O staining were performed after the corresponding inducing cultures for 3 weeks. Alizarin Red staining was performed after osteogenic cultures for 2 weeks.
