# Supplementary figures and images for: Spironolactone Ameliorates Senescence and Calcification by Modulating Autophagy in Rat Tendon-Derived Stem Cells via the NF-κB/MAPK Pathway
Source: Oxid Med Cell Longev. 2021 Jun 30;2021:5519587. doi: 10.1155/2021/5519587 (PMC8263237; doi:10.1155/2021/5519587)

NC

SP(10  $\mu$ M)

Alizarin red

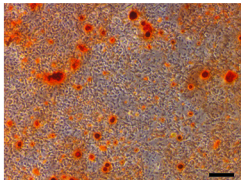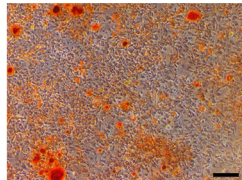

Safranin O

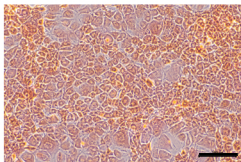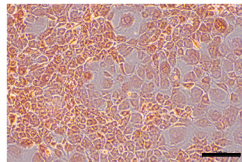

Oil red

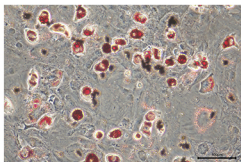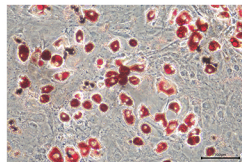

Supplement: Supplementary Materials — Supplementary Figure: SP has no significant effect on TDSC trilineage differentiation. SP with the concentration of 10 μM/mL. Safranin O staining and Oil Red O staining were performed after the corresponding inducing cultures for 3 weeks. Alizarin Red staining was performed after osteogenic cultures for 2 weeks. [file 5519587.f1.pdf]
